# Using small molecules as a new challenge to redirect metabolic pathway

**DOI:** 10.1007/s13205-013-0185-6

**Published:** 2013-11-30

**Authors:** Dina Morshedi, Farhang Aliakbari, Hamid Reza Nouri, Majid Lotfinia, Jafar Fallahi

**Affiliations:** 1 Department of Industrial and Environmental Biotechnology, National Institute of Genetic Engineering and Biotechnology, Shahrak-e Pajoohesh, km 15, Tehran-Karaj Highway, P. O. Box: 14965/161, Tehran, Iran; 2 Department of Biotechnology, Semnan University of Medical Sciences, Semnan, Iran; 3 Department of Stem Cells and Developmental Biology, Cell Science Research Center, Royan Institute for Stem Cell Biology and Technology, ACECR, Tehran, Iran; 4 Department of Biochemistry, Pasteur Institute of Iran, Tehran, Iran

**Keywords:** Acetate, *Escherichia coli*, Butyric acid, Lithium chloride, Propionic acid, Recombinant protein

## Abstract

The presence of acetate in the bacterial medium leads to a reduction in the growth rate of cells and recombinant protein production. In this study, three compounds including propionic acid, lithium chloride and butyric acid were added to the medium which decreased acetate levels and enhanced recombinant protein production (alpha-synuclein). In fact, propionic acid and lithium chloride are both known as acetate kinase inhibitors. The results obtained in the case of butyric acid were similar to those of the two other compounds indicating that butyric acid may act through a mechanism similar to propionic acid and lithium chloride. Consequently, it was shown that the presence of each of these supplements (5–200 μM) increased recombinant alpha-synuclein production and cell density by approximately 10–15 %. HPLC analysis showed that the levels of acetate in the media containing the supplements were considerably less than those of the control. Furthermore, pH values remained almost constant in the supplemented cultures. Growing the bacteria at lower temperatures (25 °C) indicated that the positive effects of these supplements were not as effective as at higher temperatures (37 °C), presumably due to the adequate balance between oxygen and carbon consumption. This study can confirm the viewpoint regarding the harmful effects of acetate on the recombinant protein production and cell density. Besides, such methods represent easy and complementary ways to increase target recombinant protein production without negatively affecting host cell density, and requiring complex genetic manipulation.

**Electronic supplementary material:**

The online version of this article (doi:10.1007/s13205-013-0185-6) contains supplementary material, which is available to authorized users.

## Introduction

A number of bacteria, particularly *Escherichia coli*, are common hosts for the expression of a wide variety of recombinant proteins associated with therapeutic, diagnostic and industrial applications. However, in the case of *E. coli*, one of the problems during its growth is the reduction and sometimes elimination of recombinant protein production. One of the main reasons for these unfavorable outcomes is the generation of acetic acid as a harmful by-product (Eiteman and Altman 2006; Pflug et al. 2007). During glucose consumption, bacteria release acetate into the medium. It is believed that acetate can have different undesirable effects on bacterial growth and productivity. A rising amount of acetate in the medium causes inhibition of cell growth (Jin et al. 2012; Luli and Strohl 1990; Roe 2002). In fact, the presence of acetate has negative effects on recombinant protein production (Jensen and Carlsen 1990), and by making the environment more acidic might influence bacterial growth (Desvaux 2006; Richmond et al. 2012). In addition, it has been shown that acetate causes the plasmid copy number in the host to drop off considerably (Cunningham et al. 2009; Pan et al. 2010). There has been much interest in how acetate accumulation can have broad negative effects, such as reduction in the pH of the culture medium. Another effect is the deficiency of certain essential amino acids like methionine (Roe 2002) or nucleic acid sources (Cunningham et al. 2009; Pan et al. 2010).

There are currently many efforts being attempted to block the formation of acetate and its release into the media. Industrial strategies tend toward reducing acetate production by the modification of external or internal parameters connected to acetate production. Some of these methods have achieved reduction in acetate production and subsequent increase in recombinant protein production (Aristidou and San 1995; De Anda et al. 2006; Vemuri et al. 2006). The external parameters known as “process controlling” include medium modification, limitation of glucose consumption as well as aeration. For example, controlling glucose consumption rate by complex glucose feeding schemes has successfully reduced acetate accumulation in *E. coli* cultures (Akesson et al. 2001; Phue et al. 2005; Shiloach et al. 1996).

The internal genotype of the host cell can also be altered (De Mey et al. 2007; Papagianni 2012). Some of these approaches include engineering strains to modify the glucose uptake rate (glucose phosphotransferase system *ptsG*) (De Anda et al. 2006; Knabben et al. 2011), redirecting the carbon flux toward less inhibitory by-products (e.g., acetoin by acetolactate synthase *als*) (Aristidou and San 1995), ethanol production through the pet operon (Diaz-Ricci et al. 1992; Ingram and Conway 1988), storage of excess carbon as glycogen (Dedhia et al. 1994) and elimination of the major acetate formation pathway (acetate kinase *ackA*, phosphotransacetylase *pta*) (De Mey et al. 2007; Phue et al. 2010).

The objective of this study was to modify the medium content in a simple and moderate way to improve recombinant protein (alpha-synuclein) production without negative effects on cell density. Accordingly, propionic acid, butyric acid and lithium chloride were added to the culture media to observe their impact on recombinant protein production and acetate reduction. In fact, propionic acid and lithium chloride are known to act as acetate kinase inhibitors (Fox and Roseman 1986). And, on the other hand, it was assumed that butyric acid might act as an acetic acid analogue and thus affect acetate production. We just focused on recombinant protein production and acetate reduction by these compounds. Consequently, the effects of the above mentioned compounds on the amount of acetate released into the medium, cell density, pH of the culture medium and also recombinant protein production were examined. Alpha-synuclein was expressed as a recombinant protein to examine the quality of the expression. This protein is known to be associated with neurodegenerative diseases. It has been produced in vitro for several years to study the mechanism of amyloid formation, and screen fibril inhibitors, or as a biomaterial in nanobiotechnology. Alpha-synuclein can enter into the preplasmic space of *E. coli* without the host specific signal peptides (Huang et al. 2005). We explored the negative effects of acetate on this preplasmic protein in the presence or absence of the mentioned compounds.

## Materials and methods

### Biochemicals and reagents

Most of the biochemicals and reagents used in this study were obtained from Merck (Germany) and were of analytical grade or higher. Isopropyl β-d-thiogalactopyranoside (IPTG), kanamycin and chloramphenicol were purchased from Sigma-Aldrich (USA).

### Plasmid and strain

The expression plasmid pNIC28-Bsa4 (7,284 bp) containing the human α-synuclein cDNA and a kanamycin resistance gene carrying the T7-lacO promoter were transformed into *E. coli* BL21 (DE3)-pLysS.

### Expression of recombinant α-synuclein

The transformed cells were screened on Luria–Bertani Agar (LB) medium containing kanamycin (50 μg/mL) and chloramphenicol (34 μg/mL). An overnight culture derived from a single colony of the transformed *E. coli* was prepared in LB broth containing the same concentrations of the antibiotics. Terrific broth (TB) was used for the expression of the protein, and was based upon tryptone (12 g/L), yeast extract (24 g/L), KH_2_PO_4_ (2.3 g/L), K_2_HPO_4_ (12.5 g/L), and glucose (10 g/L) instead of glycerol. Subcultures at a dilution of 1/50 from an overnight starter were prepared and incubated at temperatures of 37 and 25 °C with shaking at 200 rpm. When the optical density (OD_600_) approached 0.6–0.7, protein expression was induced using isopropyl β-d-thiogalactopyranoside (IPTG) at the final concentration of 500 μM, and the cells were then grown overnight (approximately 15–16 h), under the same conditions mentioned above. Sampling was carried out 7 h after induction and overnight cultivation. All samples were centrifuged at 5,000*g* for 10 min. The pellets were then used for the analyses of recombinant protein production.

### Addition of supplements

Propionic acid, at different concentrations of 5, 10, 20 and 200 μM, was added to the individual cultures at the two stages of inoculation and induction. Lithium chloride and butyric acid were also added to individual cultures at the same mentioned stages. TB medium with no supplementation of propionic acid, lithium chloride and butyric acid was used as control.

### Cell density measurement

To measure cell density, the optical density of culture samples was recorded at 600 nm (OD_600_) using a Beckman DU 500, UV–visible spectrophotometer (USA). Samples were diluted with TB medium to enable photometric measurement in the linear range between 0.1 and 0.5 OD. One unit of OD_600_ corresponds to a dry cell weight of 0.34 g/L.

### Estimation of protein concentrations

Protein expression was examined by sodium dodecyl sulfate polyacrylamide gel electrophoresis (SDS-PAGE). Protein samples were loaded onto a 12 % SDS–polyacrylamide gel and the percentage of recombinant alpha-synuclein production was analyzed by the AlphaEase FC image analysis software, version 6.0.0. Western blotting was carried out with the alpha-synuclein monoclonal antibody (Amersham Biosciences, UK).

### Analysis of acetate using high-performance liquid chromatography

Acetate accumulation in the culture media was determined by high-performance liquid chromatography (HPLC) using a Cecil 4200 HPLC system (CE 4200 England) equipped with an ODS-3 C18 column (MZ, Germany). The mobile phase consisted of acetonitrile—ddH_2_O (9/1) plus 0.1 % H_3_PO_4_, and a flow rate of 0.5 mL/min was applied without any gradient application. An injection volume of 20 μL was applied and detection of acetate was carried out at a wavelength of 215 nm using a UV–visible detector. A standard curve was prepared using different concentrations of acetate (0–20 μg in 20 μL) that were diluted in the same mobile phase as shown in Supplementary Fig. 1A. The retention times of propionic acid and butyric acid were less than that of acetic acid, as shown in Supplementary Fig. 1B.

### Assessment of pH

The pH values of samples from overnight cultivations in the presence and absence of propionic acid were analyzed by the Beckman Phi 72 pH meter.

### Statistical analysis

All experiments were repeated three times. The data obtained were analyzed for significant differences between the control and experimental groups using the SPSS software version 16.0, involving the unpaired Student’s *t* test. *P*_value_ <0.05 was considered as significant.

## Results

### Effect of propionic acid on recombinant protein production and cell density

**Fig. 1:**
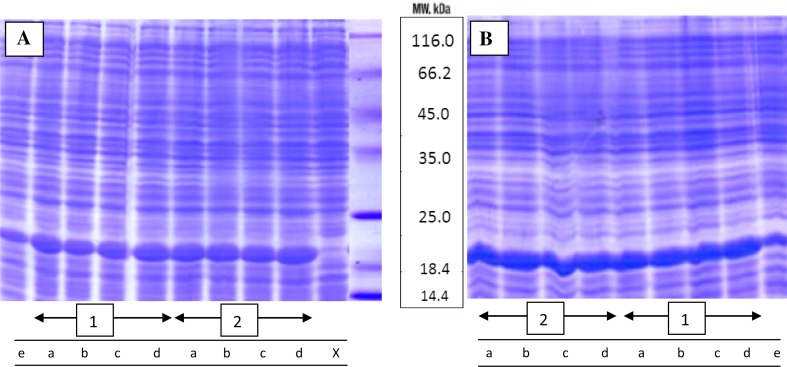
SDS-PAGE pattern of total proteins. SDS-PAGE pattern in the presence of propionic acid at different concentrations (*a* 5 μM, *b* 10 μM, *c* 20 μM and *d* 200 μM), as compared to the control (*e*) which were added at inoculation (*1*) and (*2*) induction times, 7 h of incubation (**a**) and overnight cultivations (**b**). *x* before induction

**Fig. 2:**
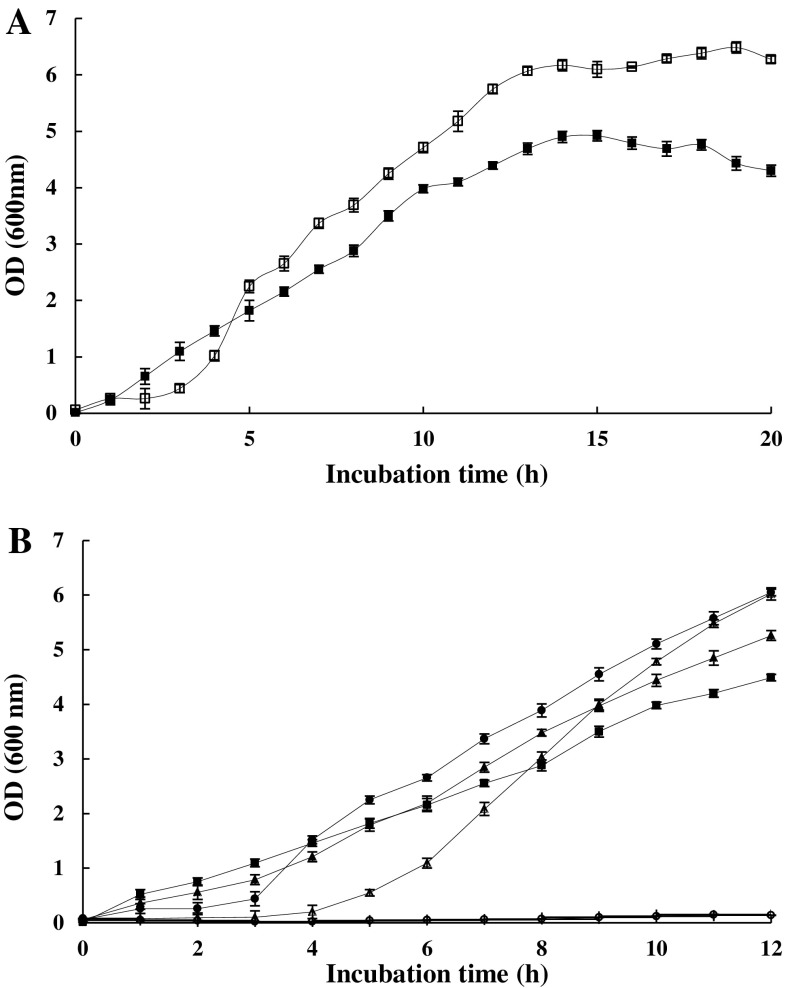
(**a**) Growth rate in the absence (*filled square*) and the presence of 200 mM propionic acid (*unfilled square*). (**b**) Growth rate in the presence of different concentrations of propionic acid that include: 0 μM (*filled square*), 20 μM (*filled triangle*), 200 μM (*filled circle*), 500 μM (*unfilled triangle*), and 4 mM (*unfilled circle*). Propionic acid was added at the time of inoculation. The samples were grown at 37 °C in shaking flasks. The stated errors are the SDs of three repeats

As mentioned previously, cell density and protein production in *E. coli* could be amplified by reducing the accumulation of acetate in the bacterial culture medium (Fox and Roseman 1986). Accordingly, this study investigated whether adding the aforementioned compounds to the culture medium could reduce acetate accumulation and subsequently enhance recombinant protein production. Initially, the effects of propionic acid as an acetic acid analogue and acetate kinase competitive inhibitor were investigated (Fox and Roseman 1986). The effect of propionic acid on recombinant protein production was verified using shake flask cultivations. Figure 1 shows the polyacrylamide gel electrophoresis patterns of protein in the presence of different concentrations of propionic acid (5, 10, 20 and 200 μM), after 7 h of growth (a) and overnight (b) cultivation. Cultures were supplemented with propionic acid in two steps (inoculation and induction times). Western blotting also confirmed that the protein was alpha-synuclein, as indicated in Supplementary Fig. 2A. Supplementary Fig. 3 which is derived from Fig. 1 indicates the percentage of recombinant protein production as estimated by the AlphaEase FC image analysis software. The production of alpha-synuclein in the presence of propionic acid increased by up to 9 % after 7 h of growth and 12 % following overnight cultivation, when compared to that of the control at 37 °C. Cell density also rose in the presence of propionic acid. The growth rate and the biomass of the cultivation were determined using optical density (OD) measurements at 600 nm. As shown in Fig. 2a, an increase in OD was observed for the samples treated with 200 μM of propionic acid, when compared to that of the control, especially regarding the overnight cultivation (approximately 15–16 h). Figure 2b shows the effects of different concentrations of propionic acid on the growth curve. It seems that the presence of propionic acid at the different concentrations has significant effects on both the cell density and protein production. In fact, by increasing the amount of propionic acid, a longer lag phase is observed. Furthermore, in the presence of high levels of propionic acid, cell growth is completely suppressed.

### Effects of propionic acid on acetate production

**Fig. 3:**
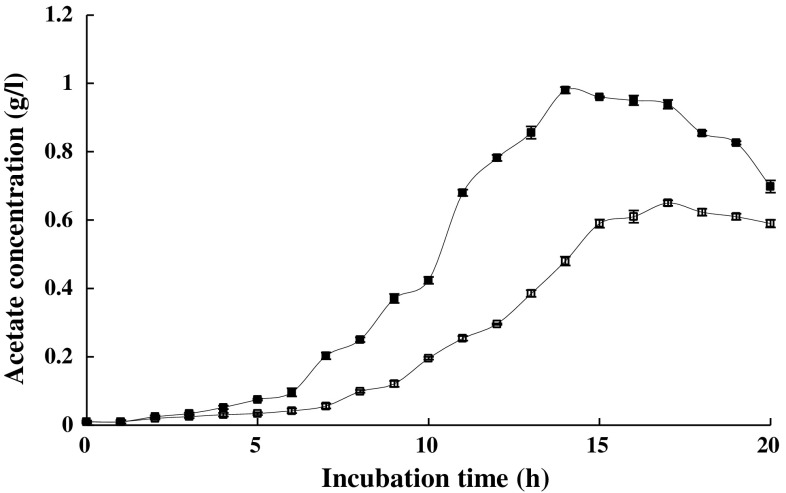
Acetate concentration in a 1 L medium in the absence (*filled square*) and the presence (*unfilled square*) of 200 μM propionic acid. Propionic acid was added at the time of inoculation. The stated errors are the SDs of three repeats

Factors, such as changing the balance between glucose metabolism and oxygen consumption, cause an increase in acetate accumulation during cultivation. Considering that propionic acid enhanced cell density and protein production, it was thus assumed that this carboxylic acid may have an impact on acetate accumulation. Therefore, using HPLC, as mentioned briefly in the materials and methods, acetate accumulation was monitored during cultivation in the presence of propionic acid at the concentration of 200 μM. Figure 3 demonstrates the kinetics of acetate production in the presence of 200 μM propionic acid. In culture media supplemented with 200 μM propionic acid, acetate production decreased by 27.5 and 49.5 % following 7 h of growth and overnight (approximately 15 h) cultivation, respectively. According to the kinetics of acetate production in the presence of 200 μM propionic acid, a similar effect was also expected in other concentrations, after 7 and 15 h of cultivation (Supplementary Table 1) Fig. 3 and Supplementary Table 1 show that acetate production increased in the control medium throughout cultivation, whereas the addition of propionic acid to the medium had negative effects on acetate accumulation.

### Effects of propionic acid on medium acidification

**Fig. 4:**
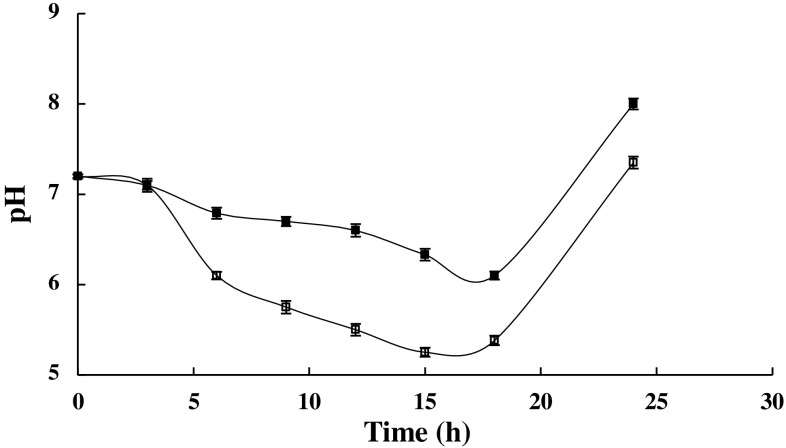
Fluctuations in pH in the presence (*dark filled*) and the absence of propionic acid (*white filled*). 200 μM propionic acid was added at the time of inoculation. The stated errors are the SDs of three repeats

Release of acetate into the medium causes a change in the pH of the medium, therefore, in this study, the pH values of the culture media in the presence and absence of propionic acid were analyzed throughout growth. Results showed that pH of the culture medium supplemented with this compound remained almost constant throughout growth when compared to that of the control. As expected, the pH values in the control medium decreased significantly (Fig. 4), thus confirming the release and accumulation of acetate in the medium.

### Effects of butyric acid and lithium chloride on recombinant protein production, cell density, acetate production and medium acidification

**Fig. 5:**
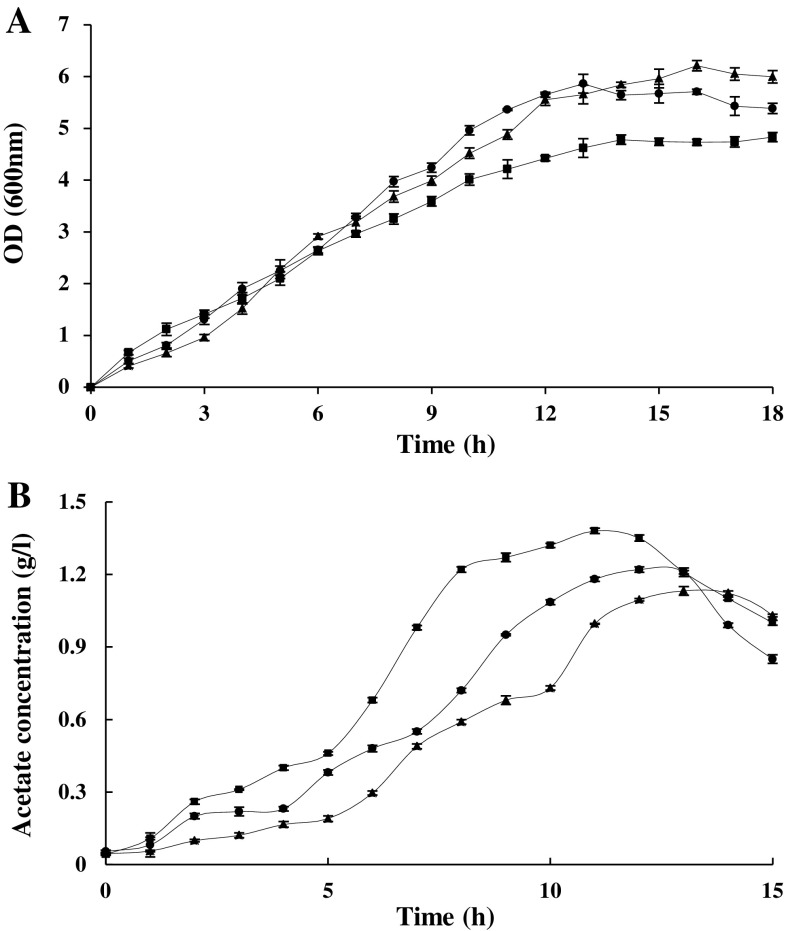
Changes in OD_600_ (**a**) and acetate concentration (**b**) of the cultures in the absence (*filled square*) and the presence of lithium chloride (*filled circle*) and butyric acid (*filled triangle*). 20 μM of lithium chloride and butyric acid were added at the time of inoculation. The stated errors are the SDs of three repeats

Other compounds that may have similar effects to that of propionic acid were also investigated. One of the candidates was butyric acid which was considered as an analogue of acetic acid and assumed to interrupt acetate release, thus paving the way for high production of recombinant protein. Despite previous studies that demonstrated butyric acid could not function as an analogue during acetate metabolism in *E. coli* (Fox and Roseman 1986), this investigation revealed that it can have the same effect as propionic acid on the acetate pathway in *E. coli*. Another candidate was lithium chloride which has been known to influence biological systems in different ways, including inhibitory effects on the enzyme acetate kinase (Fox and Roseman 1986). This research showed that both lithium chloride and butyric acid could also have positive effects, similar to that of propionic acid, regarding recombinant protein production. The effects of two concentrations of butyric acid and lithium chloride on protein production are demonstrated in Supplementary Figure 4. Similar to propionic acid, these two compounds were also added to the culture medium in two steps during growth. The percentage of recombinant protein production was enhanced by up to 8–12 % after 7 h of growth and 8.6–12.9 % following overnight cultivation (Supplementary Fig. 4). Supplementary Figures S2B and C show the results of the Western blotting procedure which confirmed that the protein was alpha-synuclein. Figure 5 demonstrates changes in OD (600 nm) and acetate concentration of the cultures in the presence and absence of lithium chloride and butyric acid (20 μM at inoculation time). Results show that cell density had almost increased in the presence of lithium chloride and butyric acid and acetate production had decreased.

As shown in Fig. 5a, an increase in the OD of samples treated with lithium chloride and butyric acid was observed relative to the control; especially regarding the cultivation of cultures involving approximately 13–16 h of incubation. In fact, cell density was increased by up to 21.16 and 23.8 %, in the presence of lithium chloride and butyric acid, respectively. Furthermore, acetate accumulation was measured during growth in the presence of the abovementioned supplements. Figure 5b shows that acetate production was reduced following the addition of lithium chloride and butyric acid, when compared to that of the control. Acetate accumulation was decreased up to 40.98 and 48.36 %, in the presence of lithium chloride and butyric acid, respectively. HPLC analysis showed that in the presence of these supplements (either added at the time of inoculation or induction) there was a decrease in acetate accumulation. In the presence of lithium chloride and butyric acid, the amount of acetate released into the medium decreased to 46.45 and 32.59 %, respectively, in approximately 7–13 h. Furthermore, the addition of these two compounds to the culture media did not change the pH values of the media as much as those observed in the control medium during cultivation (data not shown).

In fact, propionic acid and lithium chloride are known to act as acetate kinase inhibitors (Fox and Roseman 1986). And, on the other hand, it was assumed that butyric acid might act as an acetic acid analogue and thus affect acetate production.

 As stated above, propionic acid and lithium chloride are identified to act as acetate kinase inhibitors (Fox and Roseman 1986) and their effects on decrease of acetate levels and enhancement of protein production almost certainly due to their functions as acetate kinase inhibitors, and butyric acid might have the similar role on the acetate accumulation. In this regard, more investigation was carried out concerning the effects of butyric acid on the cell density and recombinant protein production including the addition of more concentrations of such compound to the culture medium and provided more evidences to support the hypothesis. Supplementary Figure 5 showed the SDS-PAGE pattern of the total proteins in presence of different concentrations of butyric acid (10, 50, 200, 400 μM). Supplementary Figure 6 depicts the growth rate of bacteria in the absence and the presence of different concentrations of butyric acid (200, 400, 4,000, 10,000 μM) compared to that of control. As elucidated from this Figure, in spite of propionic acid, the higher concentrations of butyric acid (4 and 10 mM) did not have negative effects on growth rate, however, it inspired the lag phase to become longer. In this concern, it is worth mentioning that butyric acid smells terrible and it is hard to work with it. In addition it is not necessary to use the higher amount of such compound. Furthermore, lithium chloride is involved with several pathways in bacteria, so we concluded that propionic acid was a better compound and effortless to work. Therefore, the focus of this study is more on the propionic acid and the results for two other compounds support the idea that such compounds can decrease acetate accumulation and enhance recombinant protein production.

### Effect of incubation conditions

Changing the incubation condition, such as temperature or speed of agitation during cultivation can highly affect bacterial metabolism and subsequently the growth rate and recombinant protein production. For this reason, the effects of the considered additives on acetate accumulation, recombinant protein production, pH and cell density were examined at a different temperature; 25 °C (Table 1; Supplementary Fig. 7) and agitation speed; 230 rpm after 7 h and overnight cultivations. It should be noted that the effect of temperature was investigated at a supplement concentration of 20 μM, which was added at the time of inoculation. Data obtained when using the other concentrations were found to be approximately the same (data not shown). The results indicated that cultivation of the supplemented cultures at 25 °C did not have any considerable positive effects on cell density and recombinant protein production, when compared to those obtained at 37 °C after 7 h of growth. However, following overnight cultivation at 25 °C, increases in recombinant protein production were observed. Moreover, the amount of acetate accumulation in the control medium was also found to be higher than in the supplemented samples at 25 °C (Table 1).

**Table 1:** Effects of propionic acid, butyric acid and lithium chloride on recombinant protein production, cell density, medium acidification and acetate production at 25 °C cultivations

	Protein production (%)		OD_600_		pH variation (%)		Relative acetate reduction (%)	
	7 h	ON	7 h	ON	7 h	ON	7 h	ON
Control	22.1 ± 0.25	21.6 ± 0.4	4.35 ± 0.04	4.73 ± 0.06	6.22 ± 0.03	5.78 ± 0.02		
Prop 20 μM	23.7 ± 0.1	25.2 ± 0.2	4.21 ± 0.02	4.53 ± 0.05	6.45 ± 0.04	6.17 ± 0.06	8.025 ± 0.16	11.12 ± 0.15
Bt 20 μM	23.6 ± 0.21	26.7 ± 0.26	4.09 ± 0.09	4.54 ± 0.05	6.41 ± 0.01	6.08 ± 0.07	8.24 ± 0.18	13.12 ± 0.12
Li 20 μM	22.6 ± 0.17	24.4 ± 0.23	4.41 ± 0.04	4.68 ± 0.09	6.38 ± 0.02	5.96 ± 0.05	8.02 ± 0.17	12.6 ± 0.2

All results were significantly different from the control (*P* < 0.05)

*h* hours, *ON* overnight, *Prop* propionic acid, *Bt* butyric acid; *Li* lithium chloride

**Fig. 6:**
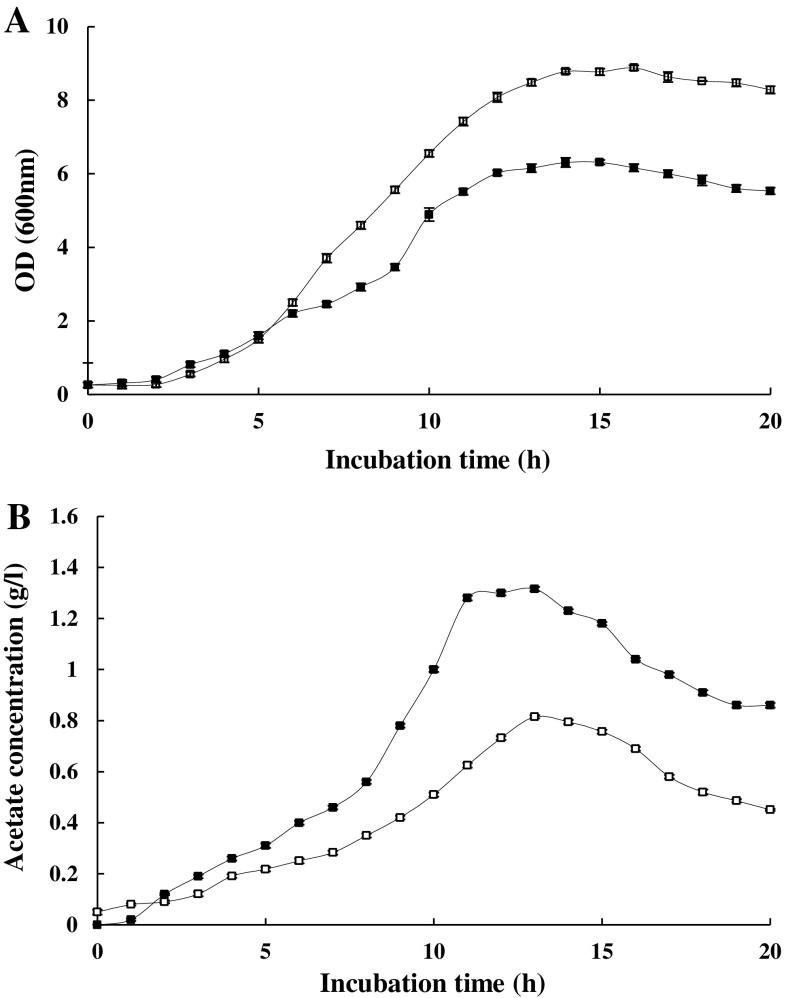
Evaluating the influence of higher cell density on the effects of propionic: (**a**) OD and (**b**) the acetate concentration of the media in the absence (*filled square*) and the presence (*unfilled square*) of 200 μM propionic acid. Propionic acid was added at the time of inoculation. The agitation speed was 230 rpm. The stated errors are the SDs of three repeats

Changing the agitation speed can have an impact on growth rate by influencing oxygen transfer through the medium (Henzler and Schedel 1991). Figure 6 depicts an increase in the OD of the cultures at higher agitation speeds and in this case the propionic acid effect is obviously highlighted. In the sample treated with 200 μM propionic acid, acetate production was decreased by 70 % and growth rate was increased by up to 27.9 %, relative to the control.

## Discussion

Propionic acid and lithium chloride have previously been identified as the inhibitors of enzymes involved in the conversion of pyruvate to acetate (Supplementary Fig. 8) (Yang et al. 1999). There are contradictions regarding butyric acid as inhibitor of such enzymes (Fox and Roseman 1986). It seems that they would be able to decrease acetate accumulation. The results of this study have also confirmed this observation.

The addition of propionic acid to the medium at concentrations ranging from 5 to 200 μM had positive effects on bacterial cell density and recombinant protein production, leading to increases of 10 and 8 %, respectively, in an overnight cultivation (approximately 13–16 h). However, this elevation in recombinant protein production is rather less than the levels obtained when using other metabolic engineering methods (Pan et al. 2010). As the findings of this study showed, the addition of propionic acid either at the time of inoculation or before induction led to similar effects and so its efficiency has been stable. The induction time was nearly 1 h after inoculation time and the results for both times were comparable. As the results of both times were equivalent, it could be possible to use such supplements during the preparation of the medium. Alteration in the culture medium allowed an increase in cell density and recombinant protein production after 7 h of incubation, and even more significant results were obtained following overnight cultivation. In the control samples, however, the percentage of heterologous proteins against total proteins reduced dramatically after overnight incubation. There is an assumption that long-time incubation may lead to destabilization of plasmids, because of lower levels of carbon being available for nucleic acid and amino acid precursors (Cunningham et al. 2009). However, by inhibiting consumption of the carbon supply in unwanted products like acetate, the stability of plasmid becomes presumably enhanced (Cunningham et al. 2009). In this study, the production of the recombinant protein in the control samples was dramatically decreased. Nevertheless, protein production in supplemented cultures remained considerably stable during long-term cultivations. Study of the kinetics of acetate production revealed that there is a significant reduction in acetate accumulation in the media of the treated cultures, and this was highlighted approximately during 11–18 h of cultivation.

Furthermore, butyric acid was found to have effects similar to those of propionic acid and lithium chloride, with regard to cell density and recombinant protein production. The present results suggest that these compounds may act in a similar way on acetate accumulation and consequently recombinant protein production. The HPLC data showed that in their presence, the amount of acetate decreased remarkably, confirming this hypothesis.

Reductions in the pH of the culture medium as well as in the cytoplasmic space have been considered as some of the factors responsible for the negative effects of acetate (Desvaux 2006; Richmond et al. 2012). Protons can interact with membranes or diffuse across membrane bilayers and induce anion accumulation (Booth 1985; Stratford and Anslow 1998). The presence of propionic acid, lithium chloride or butyric acid showed that addition of the supplements protected culture media from extraordinary fluctuations in pH value.

Acetate formation is the result of a disturbed balance between oxygen consumption and aerobic glucose metabolism, so the excess carbon flux from glucose or other compounds leads to the repression of the TCA cycle enzymes, and the promotion of uncoupled metabolisms (El-Mansi 1989; Majewski and Domach 1990). The production of acetate represents a loss of carbon flux to cell growth as well as a loss of recombinant protein production. It has been demonstrated that the metabolic engineering methods which cause the inhibition of acetate production, have extremely positive effects on plasmid stability and recombinant protein production (Cunningham et al. 2009; Pan et al. 2010).

On the other hand, some results from the metabolic analysis of carbon redistribution have shown that the *ackA*–*pta* mutation or antisense RNA systems reduce acetate levels at the expense of cell density (Kim and Cha 2003; Yang et al. 1999). However, in the experiments of this study, cell densities did not reduce and also fairy improved. In addition, in the *ackA*–*pta* deficient strain, a much higher rate of lactate formation, and simultaneously, lower rates of formate and ethanol synthesis have been observed (Kim and Cha 2003; Knabben et al. 2011). In other studies, researchers have succeeded in increasing cell biomass dramatically by inducing glycogen formation from excess carbon via using certain other mutations or transforming cells with specific plasmids (Dedhia et al. 1994).

When *E. coli* cells are growing under anaerobic conditions, sugars are fermented to a variety of products (Aristidou and San 1995; Clark and Cronan 1980; Ni et al. 2011). These pathways are dominant in the bacteria which use carbohydrates as sources of energy (Desvaux 2006; Zhang et al. 2012). One important pathway involves the conversion of acetyl-CoA to acetaldehyde by means of acetyl-CoA dehydrogenase. The acetaldehyde is subsequently converted to ethanol by alcohol dehydrogenase. Adding propionic acid, butyric acid and lithium chloride perhaps reduces the carbon flux to acetate and to a smaller extent ethanol. Due to the aerobic process, the pathway shifts to convert the excess pyruvate into much less toxic compounds such as acetoin, lactate or TCA cycle-related compounds (Aristidou and San 1995; Wolfe 2005; Yang et al. 1999).

Considering that these compounds might interfere with other enzymes or pathways in *E. coli*, further investigations are required to clarify their functions.

As mentioned previously, when carbon metabolism changes due to a sudden burst of cell growth, the conditions of growth change, leading to a situation similar to anaerobiosis and induction of anaerobic pathways, consequently lead to increased production of acetate (Hasona et al. 2004). The high rate of glucose consumption increases the uncoupled pathways instead of the TCA cycle because of a limited amount of intermediate compounds, such as NADH and oxaloacetate (Britten 1954; Majewski and Domach 1990). The relationship between carbohydrate consumption and acetate production is common in the bioreactor-used microorganisms such as *Clostridium phytofermentans*, and acetate production can influence the manufacturing performance (Jin et al. 2012). Certain strategies have been applied to limit glucose consumption and control the balance between oxygen consumption and glucose catabolism, for example, using continuous or fed-batch fermentation (Kayser et al. 2005; Weber et al. 2005). Co-culturing with specific strains which can consume acetate as a source of energy has also shown positive effects regarding the yield of product (Zhang et al. 2013). Another way is to slow down the first phase of the bacterial growth rate by incubating cells at a lower temperature (25 °C); the growth rate would then be slower than at higher temperatures (37 °C), which can then help to balance the oxygen consumption and carbon catabolism in the aerobic pathways and the TCA cycle. Accordingly, from the results obtained for cultures grown at 37 and 25 °C in this study, it can be assumed that by slowing down the growth rate at the lower temperature, lower levels of acetate will thus accumulate in the medium. In fact, results showed that under such a condition, the addition of compounds which can inhibit acetate production did not have significant effects on bacterial growth and protein production, especially in the case of short incubation times. Conversely, in another part of this study, by increasing the agitation, propionic acid was found to be more effective regarding growth rate and protein production.

## Conclusion

In conclusion, it seems that the use of propionic acid, lithium chloride and butyric acid, which are capable of diverting metabolic pathways to decrease acetate production, is a good strategy for optimizing bacterial media and growth conditions. Thus, using such compounds decrease the carbon flux toward acetate synthesis, resulting in enhancement of recombinant protein production (alpha-synuclein) without negatively affecting host cell density. It should be noted that this effect is significant when production of acetate is high. Therefore, this strategy would be useful especially in higher cell density cultures.

## Electronic supplementary material

Below is the link to the electronic supplementary material.

Supplementary material 1 (DOCX 13 kb)

Supplementary material 2 (DOCX 68 kb)

Supplementary material 3 (DOCX 110 kb)

Supplementary material 4 (DOCX 20 kb)

Supplementary material 5 (DOCX 30 kb)

Supplementary material 6 (DOCX 617 kb)

Supplementary material 7 (DOCX 15 kb)

Supplementary material 8 (DOCX 143 kb)

Supplementary material 9 (DOCX 45 kb)
