# Supplementary material for: Using small molecules as a new challenge to redirect metabolic pathway
Source: 3 Biotech. 2013 Nov 30;4(5):513–22. doi: 10.1007/s13205-013-0185-6 (PMC4162896; doi:10.1007/s13205-013-0185-6)
Supplement: Supplementary file 1 — Supplementary material 1 (DOCX 13 kb) [file 13205_2013_185_MOESM1_ESM.docx]

Supplementary table 1. The acetate concentration in 1liter medium and its relative reduction at 7 and 15 h cultures in the presence and absence of propionic acid (10 and 500 µM). a: Inoculation time; b: Induction time, h: Hours;.

Given that the induction for the concentration of 500 µM propionic acid was later, the experiment for this concentration at induction time was not carried out.

|  | 7h (g/lit) | % Relative reduction (7h) | 15h (g/lit) | %Relative reduction (15h) |
| --- | --- | --- | --- | --- |
| Control | 0.234±0.08 |  | 1.1±0.1 |  |
| 10 µM a | 0.196±0.022 | 16.23 | 0.75±0. 11 | 31.81 |
| 500 µM a | 0.12±0.05 | 48.71 | 0.32±0.14 | 70.90 |
| 10 µM b | 0.188 ±0.02 | 19.65 | 0.73±0.1 | 33.63 |
| 500 µM b | ----- | ----- | ----- | ----- |

All results were significantly different from the control (P<0.05).
