# Supplementary material for: Using small molecules as a new challenge to redirect metabolic pathway
Source: 3 Biotech. 2013 Nov 30;4(5):513–22. doi: 10.1007/s13205-013-0185-6 (PMC4162896; doi:10.1007/s13205-013-0185-6)
Supplement: Supplementary file 2 — Supplementary material 2 (DOCX 68 kb) [file 13205_2013_185_MOESM2_ESM.docx]

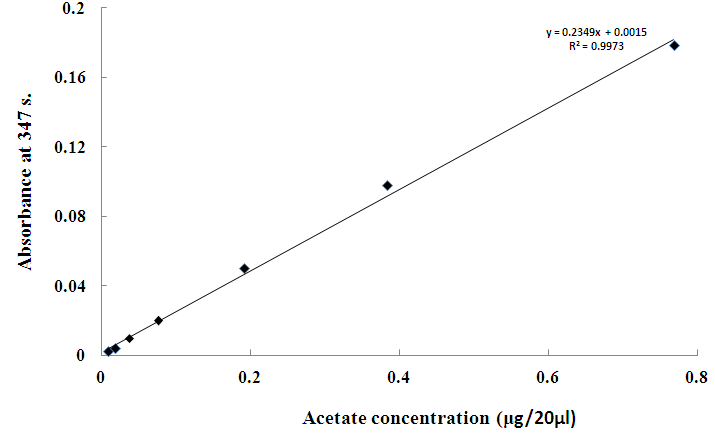


**A**


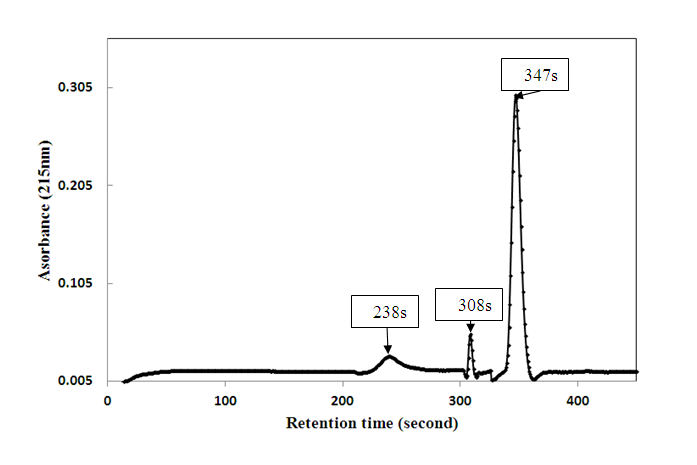


**B**

Supplementary Fig. 1. A: Overlaying of HPLC chromatogram for butyric acid (retention time: 238s), propionic acid (308s) and acetate (347s) individually, and B: standard curve of different concentrations of acetate at retention time of 347 second.
