# Supplementary material for: Using small molecules as a new challenge to redirect metabolic pathway
Source: 3 Biotech. 2013 Nov 30;4(5):513–22. doi: 10.1007/s13205-013-0185-6 (PMC4162896; doi:10.1007/s13205-013-0185-6)
Supplement: Supplementary file 3 — Supplementary material 3 (DOCX 110 kb) [file 13205_2013_185_MOESM3_ESM.docx]

**( A) (B)**


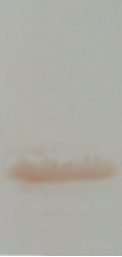

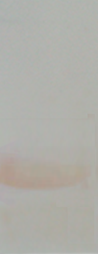

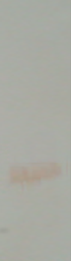

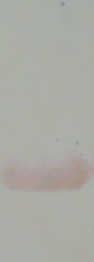


1 2 1 2

Supplementary Fig. 2. Western blotting of alpha-synuclein. A; 1: Western blotting of the protein in the TB medium, 2: Western blotting of the protein in the TB medium supplemented by propionic acid. B; 1: Western blotting of the protein in the TB medium supplemented by butyric acid. 2: Western blotting of the protein in the TB medium supplemented by lithium chloride.
