# Supplementary material for: Using small molecules as a new challenge to redirect metabolic pathway
Source: 3 Biotech. 2013 Nov 30;4(5):513–22. doi: 10.1007/s13205-013-0185-6 (PMC4162896; doi:10.1007/s13205-013-0185-6)
Supplement: Supplementary file 4 — Supplementary material 4 (DOCX 20 kb) [file 13205_2013_185_MOESM4_ESM.docx]

**B**

**A**

Supplementary Fig. 3. Measurement of alpha-synuclein production in the presence of propionic acid. Comparison of alpha-synuclein productions in the growth media supplemented with different concentrations of propionic acid at inoculation (white) and induction (black) times. 7 h (A) and overnight cultivations (B).

A
