# Supplementary material for: Using small molecules as a new challenge to redirect metabolic pathway
Source: 3 Biotech. 2013 Nov 30;4(5):513–22. doi: 10.1007/s13205-013-0185-6 (PMC4162896; doi:10.1007/s13205-013-0185-6)
Supplement: Supplementary file 5 — Supplementary material 5 (DOCX 30 kb) [file 13205_2013_185_MOESM5_ESM.docx]

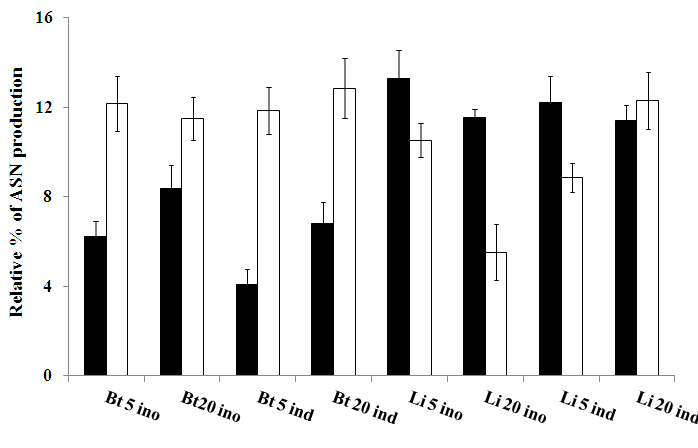


Supplementary Fig. 4. Measurement of alpha-synuclein production in the presence of lithium chloride and butyric acid. Comparison of the relative percentage of ASN production in the cultures supplemented with different concentrations (5 and 20 μM) of lithium chloride (Li) and butyric acid (Bt), to the control at inoculation (ino) and induction time (ind), after 7 h (black) and overnight cultivations (white).
