# Supplementary material for: Using small molecules as a new challenge to redirect metabolic pathway
Source: 3 Biotech. 2013 Nov 30;4(5):513–22. doi: 10.1007/s13205-013-0185-6 (PMC4162896; doi:10.1007/s13205-013-0185-6)
Supplement: Supplementary file 6 — Supplementary material 6 (DOCX 617 kb) [file 13205_2013_185_MOESM6_ESM.docx]

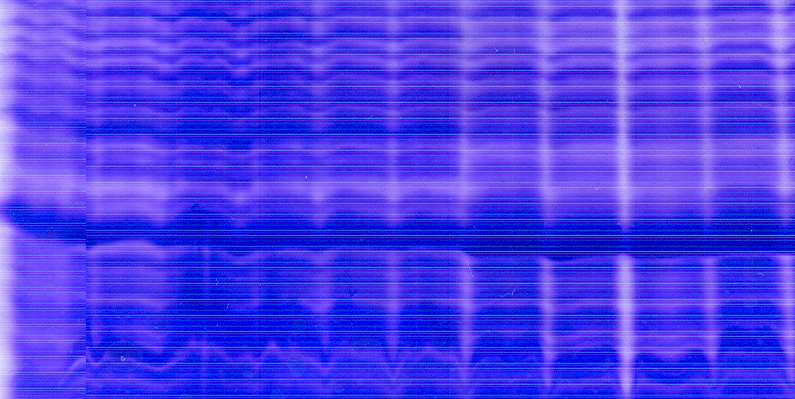


Alpha-synuclein

a b c d e f g h i j

**B**

**A**

Supplementary Fig. 5. SDS-PAGE pattern of total proteins in the presence of different concentration of butyric acid on recombinant protein production compared to the control at 7 h (A) and overnight cultivations (B), a: control (7 h); b: control (overnight) ; c: 10 μM butyric acid; d: 50 μM butyric acid; e: 200 μM butyric acid. f: 400 μM butyric acid; g: 10 μM butyric acid; h: 50 μM butyric acid; i: 200 μM butyric acid. g: 400 μM butyric acid; Cell growth was carried out at 37^◦^C in shake flasks.

1

2
