# Supplementary material for: Using small molecules as a new challenge to redirect metabolic pathway
Source: 3 Biotech. 2013 Nov 30;4(5):513–22. doi: 10.1007/s13205-013-0185-6 (PMC4162896; doi:10.1007/s13205-013-0185-6)
Supplement: Supplementary file 7 — Supplementary material 7 (DOCX 15 kb) [file 13205_2013_185_MOESM7_ESM.docx]

Supplementary Fig. 6. Growth rate in the absence (■) and the presence of different concentration of butyric acid 200 mM propionic acid: 200 μM (▲), 400 μM (□),4 mM (∆), and 10 mM. Butyric acid was added at the inoculation time. The samples were growth at 37◦C in the shaking flask.
