# Supplementary material for: Using small molecules as a new challenge to redirect metabolic pathway
Source: 3 Biotech. 2013 Nov 30;4(5):513–22. doi: 10.1007/s13205-013-0185-6 (PMC4162896; doi:10.1007/s13205-013-0185-6)
Supplement: Supplementary file 8 — Supplementary material 8 (DOCX 143 kb) [file 13205_2013_185_MOESM8_ESM.docx]

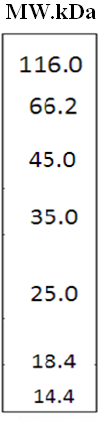


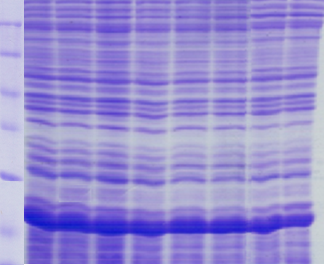


-

1

2

a b c a b c d e ***/*

Supplementary Fig. 7. The effect of temperature on the protein production. SDS-PAGE pattern of total proteins in the presence of 20 μM of the additives compared to the control at 7 h (1) and overnight cultivations (2), a: propionic acid; b: butyric acid; c: lithium chloride d: control at 7 h; e: control at overnight. Cell growth was carried out at 25^◦^C in shake flasks.
