# Supplementary material for: Using small molecules as a new challenge to redirect metabolic pathway
Source: 3 Biotech. 2013 Nov 30;4(5):513–22. doi: 10.1007/s13205-013-0185-6 (PMC4162896; doi:10.1007/s13205-013-0185-6)
Supplement: Supplementary file 9 — Supplementary material 9 (DOCX 45 kb) [file 13205_2013_185_MOESM9_ESM.docx]

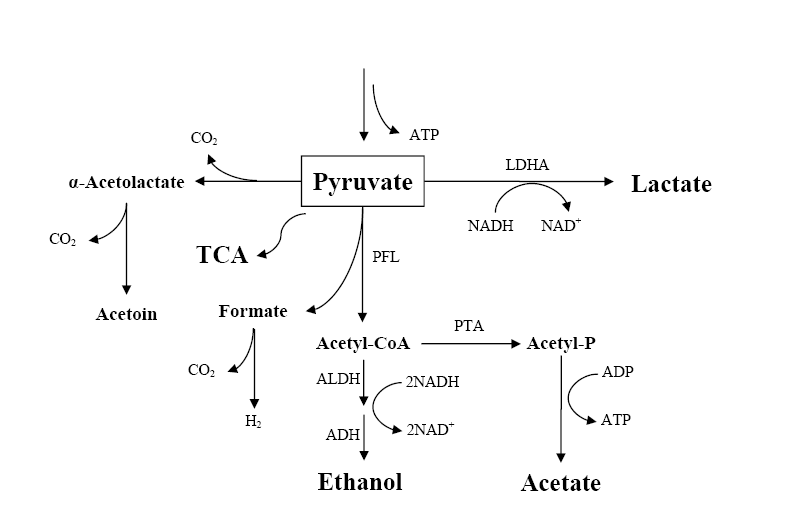


Supplementary Fig. 8. Metabolic fate of pyruvate. PFL: Pyruvate formate lyase; ACK: Acetate kinase; ALDH: Aldehyde dehydrogenase; ADH: Alcohol dehydrogenase; LDHA: Lactate dehydrogenase A; PTA: Acetyl phosphotransferase (Yang, Aristidou et al. 1999).
